# Vaginal Microbiota and Local Immunity in HPV-Induced High-Grade Cervical Dysplasia: A Narrative Review

**DOI:** 10.3390/ijms26093954

**Published:** 2025-04-22

**Authors:** Helena C. J. Schellekens, Lotte M. S. Schmidt, Servaas A. Morré, Edith M. G. van Esch, Peggy J. de Vos van Steenwijk

**Affiliations:** 1 Department of Gynecology and Obstetrics, Maastricht University Medical Centre (MUMC+), 6229 HX Maastricht, The Netherlands; 2 GROW—School for Oncology and Reproduction, Maastricht University, 6229 ER Maastricht, The Netherlands; 3 Institute of Public Health Genomics, Department of Genetics and Cell Biology, FHML, Maastricht University, 6229 ER Maastricht, The Netherlands; 4 Dutch Chlamydia Trachomatis Reference Laboratory, Department of Medical Microbiology, Infectious Diseases and Infection Prevention, Care and Public Health Research Institute (CAPHRI), Maastricht University Medical Centre (MUMC+), 6229 GT Maastricht, The Netherlands; 5 Department of Gynecology and Obstetrics, Catharina Hospital Eindhoven, 5623 EJ Eindhoven, The Netherlands

**Keywords:** vaginal microbiota, host immunity, Human Papillomavirus, cervical squamous intraepithelial lesions

## Abstract

Persistent high-risk Human Papillomavirus infection is the primary factor in cervical carcinogenesis. However, other host-related features are believed to play a role as well. Recent research suggests that the vaginal microbiome and the immune microenvironment play a significant role in the acquisition and persistence of Human Papillomavirus infection, as well as in the regression or progression of cervical intraepithelial lesions. Studies in this emerging field describe factors associated with this interaction, though the precise nature remains incompletely understood. In this narrative review, we aim to summarize the current literature on the topic and propose hypotheses and recommendations for future research and treatment strategies.

## 1. Introduction

Human Papillomavirus (HPV) is a prevalent virus that is primarily transmitted through sexual contact, with a lifetime risk of acquisition of up to 80% [1]. High-risk types of HPV (hrHPV) are oncogenetic [2], and persistent hrHPV infections are causally related to the development of premalignant anogenital intraepithelial neoplasia, including cervical low- and high-grade squamous intraepithelial lesions (cLSIL and cHSIL), and eventually progression into cervical squamous cell carcinoma [1,3,4].

Mounting evidence suggests that both the vaginal microbiome (VMB) and the host immunity play a pivotal role in HPV infection, persistence, and lesion progression. The VMB is thought to influence the host’s immune environment and vice versa [5]. The human VMB can be characterized into five community state types (CST) (Table 1) [6]. Unlike other microbiomes, such as the gut microbiome, the optimal VMB is characterized by low microbial diversity and high abundance of *Lactobacillus* (*L*.) species (spp.) as lactobacilli produce lactic acid and bacteriocin, which create a stable VMB by maintaining a low pH environment and creating a barrier against infections [7,8], inhibiting the proliferation of pathogens [9]. Several studies have been conducted to explore the interplay between CSTs and HPV infection. CST I, which is dominated by *L. crispatus*, is believed to be the most beneficial CST, and epidemiological studies have associated it with a decreased detection of HPV and cHSILs [10]. A greater diversity of bacterial species is associated with vaginal dysbiosis, such as bacterial vaginosis, which positively correlates with the risk of acquiring HPV infection and cervical lesions [11]. Species linked to dysbiosis can increase the vaginal pH [12] and reduce levels of hydrogen peroxide (H_2_O_2_) [13]. These alterations can cause disruptions of the mucosal barrier, thereby facilitating the entry of HPV capsids [14]. The mechanisms underlying the interaction between HPV and the VMB are still not fully understood. The host’s immune response within HPV-induced cervical lesions is also believed to play a crucial role in the progression and regression of cervical dysplasia [15]. An imbalance between T-helper 1 (Th1) and T-helper 2 (Th2) cells contributes to persistent hrHPV infection and the development of cervical lesions [16,17].

While current evidence suggests an interplay between HPV, the VMB, and the immune microenvironment in cervical carcinogenesis, the precise nature of these mechanisms remains incompletely understood. Therefore, the aim of this narrative review is to summarize the available literature to explore how the VMB and the immune microenvironment interact in the context of HPV-induced c(H)SILs.

## 2. Materials and Methods

**Figure 1:**
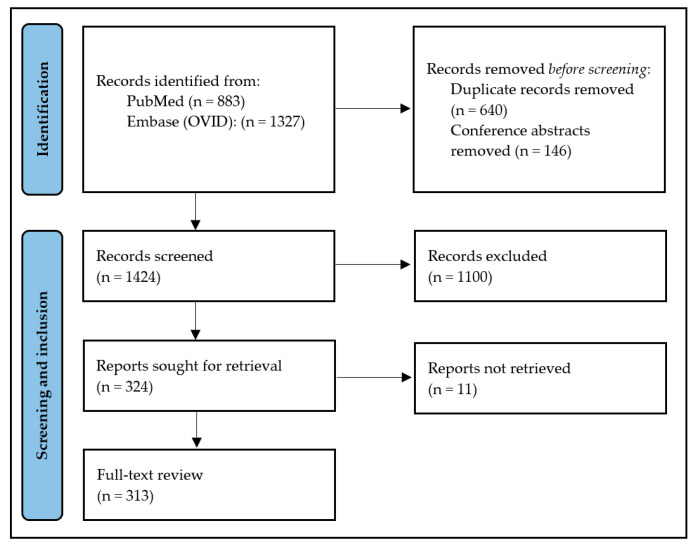
Flow diagram of review process.

The literature search was conducted using PubMed and Embase (OVID) and was last updated on 29 January 2025 (Supplementary Material S1). The results were screened by one researcher (H.C.J.S.). We included original research on the influence of the VMB on HPV infections and HPV-induced cervical lesions, as well as reviews on this topic. Additionally, we included original research and reviews on the immune environment in HPV(-induced) lesions and their connection to the VMB. Finally, we included original research and reviews on treatment modalities for cervical lesions affecting the VMB. Conference abstracts were excluded. A total of 313 articles were full-text reviewed, the most relevant findings will be discussed (Figure 1).

## 3. Results

### 3.1. The Vaginal Microbiome and Community State Types (CST)

The VMB is a complex community of microorganisms that play a crucial role in maintaining female urogenital health. In previous research, Ravel et al. divided the most common VMB compositions into five CSTs. These communities were grouped according to species abundance. CST I is dominated by *L*. *crispatus*, CST II is *L*. *gasseri*-dominated, and CST III and V are dominated by *L*. *iners* and *L*. *jensenii*, respectively. CST IV is considered the most diverse community with the highest pH [6]. CST I has the lowest median pH compared to communities dominated by *Lactobacillus* spp. other than *L*. *crispatus*. Vaginal pH is positively associated with (hr)HPV infection [18,19]. The difference in vaginal pH between CSTs suggests that the production of lactic acid might differ between species within the *Lactobacillus* genus [6]. In later research, the two isoforms of lactic acid have been studied. *L*. *crispatus* produces both the D- and L-isoform of lactic acid, whereas *L*. *iners* produces only L-lactic acid [20,21,22]. Vaginal anaerobes, on the other hand, produce cytokines and inflammatory mediators which are associated with chronic inflammation [23], and mucin degrading enzymes [24,25] that are thought to affect the mucosal layer of the cervical epithelium, reducing its ability to act as a barrier against infections [25].

Longitudinal studies have shown that the VMB is relatively stable in the majority of women [26,27,28,29,30]. VMBs dominated by *L*. *crispatus* and/or *L*. *gasseri* are generally stable, with transitions primarily occurring during menses, after which the microbiome typically transitions back. On the other hand, CST III and IV are less stable, frequently undergoing transitions between one another. These less stable CSTs are less likely to transition to the more optimal CSTs I or II [29]. Women with VMB dominated by *L*. *crispatus* are less likely to transition to bacterial vaginosis (BV)-associated VMB than women with *L*. *iners*-dominated VMB [29,31,32].

### 3.2. The Role of the Vaginal Microbiome in HPV-Induced Lesions

As mentioned, the most important etiological factor in cervical carcinogenesis is a persistent infection with hrHPV. However, HPV alone is thought to be insufficient in causing cervical malignancies. Mounting evidence supports the causal link between vaginal dysbiosis and cervical carcinogenesis [33,34]. Research has shown that the diversity of VMB increased with advancing cervical dysplasia severity [35,36,37,38] and that an anaerobic VMB is associated with a lower regression rate of HPV-induced lesions [39].

#### 3.2.1. *Lactobacillus* Species in the Vaginal Microenvironment

Microorganisms belonging to the phylum Firmicutes, specifically *Lactobacillus* spp., are most often associated with HPV-negative outcomes [40,41] and normal cytology or histology results [42,43]. Additionally, the abundance of *Lactobacillus* spp. is negatively correlated with HPV persistence [44] and positively correlated with infection clearance [45]. The protective role of *Lactobacillus* spp. lies in its diverse functions. Lactobacilli can create a biofilm and a protective barrier by adhering to epithelial surfaces and cervicovaginal mucus, thereby inhibiting the adhesion of pathogens [46,47]. Additionally, antimicrobial compounds such as biosurfactants, H_2_O_2_, and lactic acid are produced, helping to prevent HPV from invading cervical epithelial cells [47,48]. For the synthesis of lactic acid, glycogen is essential. An increased production of estrogen leads to an accumulation of glycogen [46], which leads to cervical enrichment of *Lactobacillus* spp. [49]. A higher *Lactobacillus* spp. abundance is correlated with a lower pH [50].

The roles of different *Lactobacillus* spp. in HPV-induced cervical carcinogenesis have been studied extensively. VMB dominated by *Lactobacillus* spp. is considered to be the most beneficial community, especially *Lactobacillus crispatus*-dominated communities. It is now well established from a variety of studies that a higher abundance of *L. crispatus* is associated with (hr)HPV-negative outcomes [43,51,52] at baseline and follow-up. Additionally, it is inversely associated with hrHPV persistence [53] and the abundance of *L. crispatus* decreases with increasing severity of cervical lesions [50,54] while higher levels of *L. crispatus* at baseline decrease the risk of developing cSIL [55]. Decreased amounts of *L. crispatus* have been found in VMB with a higher alpha diversity [50] and patients with (hr)HPV infection [56], LSIL [57], HSIL [43,57,58], and even cervical carcinoma [54,57]. The protective role of *L. crispatus* can be explained by its higher H_2_O_2_ production, which has been observed in *L. crispatus*-dominated communities [50] and is known to inhibit pathogens. Additionally, as mentioned, *L. crispatus* produces both the D- and L-isoforms of lactic acid [20,21,22]. Notably, D-lactic acid levels have been found to be significantly higher in VMB containing *L. crispatus* compared to *L. iners* [59]. Lower levels of D-lactic acid have been associated with cHSIL and cervical cancer [60]. Taken together, these characteristics indicate that *L. crispatus* plays a beneficial role in the vaginal microenvironment.

While numerous studies have strongly associated *L. crispatus* with favorable outcomes, the findings regarding other *Lactobacillus* spp. are more diverse. Many studies have highlighted the protective role of *L. gasseri* in preventing cervical carcinogenesis. *L. gasseri*-dominated VMB (CST II) has been associated with normal cervical cytology [42] and HPV clearance [13,61]. Also, a decrease in the abundance of *L. gasseri* was observed in HPV-positive women [56]. However, one study reported an increase in *L. gasseri* in HPV-positive women [62]. As most studies provide evidence for the beneficial role of *L. gasseri*, while only one study highlights a possible negative effect of this species, it is reasonable to assume that *L. gasseri* is a beneficial microorganism in protecting against HPV-induced cervical lesions. Furthermore, *L. gasseri* produces both D- and L-lactic acid, which supports this assumption [60].

The findings concerning the role of *L. iners* in HPV-induced lesions are more inconsistent. While several studies have shown that a high abundance of *L. iners* (CST III) is associated with HPV-negative outcomes [43], the absence of cervical abnormalities [42], HPV clearance [63,64], and lower alpha diversity [50], as well as that a decrease in *L. iners* increases the susceptibility to HPV infection [56,65], HPV persistence [66] and higher alpha diversity [67], other studies have reported contrasting findings. Specifically, *L. iners* has been linked to (hr)HPV-positivity [68,69], slower infection clearance [13,70], and an increased risk of neoplastic progression [40,71,72,73] including cLSIL [58], cHSIL [74,75], and cervical carcinoma [76,77]. The variety of findings might be caused by the production characteristics of *L. iners*. On the one hand, *L. iners* exhibits characteristics typical of lactobacilli, but on the other hand, there are subtle differences. Lower levels of H_2_O_2_ have been observed [50], while higher levels of H_2_O_2_ are associated with defense against invading pathogens. The D-isoform of lactic acid is believed to play a protective role in cervical carcinogenesis. Research has shown that in *L. iners*-dominated communities, D-lactic acid levels are decreased, while L-lactic acid levels—and consequently the L/D-lactic acid ratio—are elevated in women with cHSIL and cervical carcinoma [60]. These characteristics contribute to a reduced capacity to maintain an acidic vaginal environment [78] and, as a result, a suboptimal vaginal pH. This in turn increases the risk of anaerobic bacteria and HPV capsids invading the epithelial cells.

While existing evidence on *L. jensenii* is more limited compared to the previously discussed *Lactobacillus* spp., contrasting results are also reported in this case. On the one hand, its enrichment is associated with the control—HPV-negative—group [79], while its reduction is linked to cervical lesions [69]; conversely, an increase in *L. jensenii* has been observed in HPV-positive women [56], in HPV persistence [66], and in cervical lesions [71]. In previous literature, the specific characteristics of *L. jensenii* have not been fully elucidated. A hypothesis for the increased association of *L. jensenii*, compared to *L. crispatus*, with HPV infection and cervical lesions could be that while *L. jensenii* shares similar characteristics, its production of antimicrobial compounds and its ability to lower the vaginal pH may be less pronounced than those of *L. crispatus*.

#### 3.2.2. Species Involved in Vaginal Dysbiosis

Genera other than *Lactobacillus* are generally associated with vaginal dysbiosis. *Lactobacillus*-depleted communities are considered risk factors for HPV acquisition [80], persistence, and cervical lesion progression [37,41,81]. When *Lactobacillus* dominance is lost, microbial diversity increases. These diverse vaginal anaerobes produce mucindegrading enzymes, which cause changes in immune and epithelial homeostasis [46]. Vaginal dysbiosis, characterized by higher alpha diversity [82], is typical of CST IV. Numerous species have been linked to a less optimal vaginal environment and an increased risk of HPV infection, and consequently, cervical carcinogenesis. The most important findings will be discussed.

Bacterial vaginosis (BV)-associated pathogens were the first to be studied in relation to HPV and cervical dysplasia. BV is an infection caused by a depletion of lactobacilli and a proliferation of *Gardnerella (G.) vaginalis* and other anaerobic bacteria and it is positively correlated with hrHPV infection [38,63,83,84] and persistence [85]. BV-related bacteria are likely related to the production of amines, which can induce oxidative stress [47], this oxidative stress is thought to promote the progression of preneoplastic cervical lesions [86]. *G. vaginalis* can adhere to the vaginal epithelium, providing a framework for the production of biofilms and the proliferation of microorganisms [87,88]. Furthermore, *Gardnerella* produces sialidases which can degrade mucosa-protecting factors, causing destruction of epithelial cells and bacterial invasion [48]. Usyk et al. showed a positive correlation of *G. vaginalis* with the progression of the disease [63].

In addition to *G. vaginalis*, other species within the Actinobacteria phylum are associated with BV and HPV. *Fannyhessea (F.) vaginae* is observed in most BV cases and is associated with a higher vaginal pH [89]. The presence of any *Fannyhessea* spp. not only increases the risk of HPV infection but it is also associated with a higher prevalence of cLSIL, cHSIL [89,90], and cervical carcinoma [54]. *Atopobium (A.)* spp., specifically *A. vaginae*, are frequently detected in (hr)HPV-positive women [91,92] and cervical lesions [55,72] including cHSIL [57,93].

Another phylum associated with BV is Fusobacteriota, with the genus *Sneathia (S.)* being most commonly enriched in (hr)HPV-positive samples [94,95] and cHSIL [36]. In a treated cohort, *S. amnii* was overrepresented before local excision of cervical dysplasia, but no longer overexpressed following treatment. This finding suggests an interaction between *S. amnii* and the development of cSIL [35]. In another study, an increased abundance of *S. sanguinegens* was linked to bacterial biofilms in vaginal fluids [96]. Additionally, *S. vaginalis* can adhere to malignant cervical epithelial cells [97], suggesting a potential influence of *S. vaginalis* on the cervical microenvironment.

*Prevotella* (*P.*) spp., belonging to the Bacteroidetes phylum, are associated with both BV and HPV as well [48,82]. *P. bivia* is considered an early colonizer in BV, it is believed that *P. bivia* paves the way for secondary colonizers, such as previously mentioned *Atopobium* and *Sneathia* [47]. *P. timonensis* is inversely associated with lesion regression [39] and positively correlated to cHSIL [56], while *P. amnii* is more commonly found in cLSIL [56]. Like *Gardnerella*, *Prevotella* spp. secrete sialidases which can contribute to HPV persistence [98]. With the presence of *Prevotella* spp., the diversity of cervicovaginal flora increases and *Lactobacillus* abundance decreases [36,49].

To conclude, BV-associated bacteria are commonly enriched in HPV-positive patients [95] and are associated with a slower infection clearance rate [13] and HPV persistence [94]. These bacteria are negatively correlated with the presence of *L. crispatus*, yet exhibit symbiotic relationships with each other [54,99,100].

While extensive research has been conducted on the role of BV-associated bacteria in HPV-induced lesions, other bacteria are associated with CST IV, and vaginal dysbiosis, as well. The most commonly described genera within the Firmicutes phylum are *Streptococcus*, *Mycoplasma*, *Shuttleworthia*, and *Dialister*. These have been associated with (hr)HPV positivity [52,56,101,102], infection persistence [100], cLSIL [36], cHSIL [56,74,103], and cervical carcinoma [36,49,54]. *G. vaginalis* primarily drives peptidoglycan biosynthesis. However, *Streptococcus* spp. contribute to this process [90]. Peptidoglycan biosynthesis has been linked to the development of cervical cancer [104], likely because peptidoglycan can be recognized by the immune system as a sign of bacterial infection. This can trigger inflammation, and chronic inflammation is a known risk factor for the development of malignant lesions.

*Pseudomonas* spp., within the Proteobacteria phylum, have been found in communities of women infected with (hr)HPV [49]. Persistent infection [100,105] and ensuing cervical lesions [43,106], including cHSIL [103], have been linked to this bacterium. However, another study found a decrease in *Pseudomonas* spp. as the severity of lesions progressed [107].

The Tenericutes phylum has been associated with HPV-positive outcomes. Specifically, *Ureaplasma (U.)* spp. are linked to hrHPV infections [92,101,108,109] and cervical dysplasia [110]. In contrast, *U. urealyticum* has been positively correlated with infection clearance [51].

*Trichomonas (T.) vaginalis*, a species within the Metamonada phylum, is positively correlated with hrHPV infection and an increased risk of cervical dysplasia development [111]. This association may be explained by *Trichomonas* competing with lactobacilli for glycogen in vaginal epithelial cells, which inhibits the growth of *Lactobacillus* spp. and leads to an increased vaginal pH [112].

Research showed a positive association between HPV infection, cSIL, and *Chlamydia (C.) trachomatis* [34,38,49,113]. This increased chance of HPV acquisition is likely a consequence of the disruption in the cervical epithelium caused by *C*. *trachomatis*, increasing the chance of HPV entry to the basal epithelium [114,115,116,117].

In summary, bacteria that characterize the diversity of CST IV are significantly associated with (hr)HPV infection, persistence, and progression to cervical dysplasia and even cervical carcinoma. CST IV is generally linked to a higher vaginal pH, which increases the susceptibility to pathogen invasion and decreases *Lactobacillus* spp. dominance. No significant differences in the VMB composition immediately prior to and immediately after regression of CIN 2 were observed, suggesting that cervical dysplasia may not be driving the VMB but that the VMB itself could be a risk factor for the development of cSIL [39]. While HPV has been shown to influence the composition and diversity of the VMB, BV-associated bacteria can further reinforce this trend [107]. In contrast, some CST IV-related bacteria have also been found in HPV-negative cases and HPV clearance [51,107], suggesting that the relationship between CST IV and HPV infection is not straightforward. This implies that other factors, such as the immune microenvironment, play a role in this interplay.

### 3.3. The Immune Microenvironment in HPV-Induced Lesions

HPV infection can alter the host immune response [118], disrupting both innate and adaptive immunity, leading to persistent infection and cSIL development [119]. HPV-induced changes in immunity could potentially create an environment that facilitates the growth of pathogenic microorganisms, further driving inflammation and establishing a positive feedback loop [46,120], which ultimately promotes the development of cervical lesions [120].

B and T lymphocytes are key elements in the anti-tumor immune response. T lymphocyte-mediated cellular immunity can inhibit the growth of tumor cells [121,122,123]. The anti-tumoral characteristics of T cells consist of CD4+ T helper cells and CD8+ cytotoxic T cells. A decrease in the CD4+/CD8+ ratio indicates that the immune response is impaired [124,125]. Wang, et al. observed that the systemic CD4+ T cell count and CD4+/CD8+ ratio of patients infected with hrHPV were significantly lower compared to the control group [126]. Th1 cells can activate cellular immunity and release pro-inflammatory cytokines including interferon-gamma (IFN-γ), interleukin (IL)-1β, IL-2, IL-6, IL-8, tumor necrosis factor (TNF)-α, and TNF-β. IL-1β is associated with tumorigenesis, angiogenesis, metastasis, and an increased risk of progression to cervical carcinoma in women with cHSIL [78]. IL-6 is believed to promote cell proliferation and inhibit apoptosis. High levels of IL-6 could stimulate Vascular Endothelial Growth Factor (VEGF) production and promote cervical carcinogenesis [127]. IL-8 is crucial in transporting immune cells to the inflammation site [78]. Th2 cells regulate humoral immunity, and regulatory T cells (Tregs) are important in immunosuppression. Both produce anti-inflammatory cytokines IL-4 and IL-10. IL-4 maintains tissue health by regulating the immune response and indirectly impacting the microbial community composition [78]. CD4+ T cells can differentiate into Th17 cells, which produce IL-17. IL-17 promotes inflammation, which can suppress the effectiveness of the immune response, potentially leading to an ineffective defense against HPV [128].

As reviewed by Muntinga et al., the host’s immunity within HPV-induced cervical lesions plays a crucial role in the progression and regression of cervical dysplasia [15]. In cHSIL, there is an increase in immature dendritic cells (iDCs), Tregs, programmed death-ligand (PD-L1+) cells, and macrophages, while the levels of Langerhans cells (LCs), CD4+, and CD8+ T cells decrease compared to both healthy cervix and low-grade lesions [15]. In women with (pre)malignant cervical lesions, lower serum concentrations of IFN-γ and higher serum concentrations of IL-10 were found. Thus, suggesting that an imbalance between Th1 and Th2 cells may lead to persistent HPV infection and lesion progression [16,17]. In another study, the Th1/Th2 ratio in cervicovaginal lavages was decreased [129]. As the severity of cervical lesions increased, concentrations of IL-10, SIgA, and IgG were elevated, while IL-2 levels were decreased in cervicovaginal lavages [129]. In patients with HPV clearance, more endocervical LCs were observed than in persistence and HPV-negative controls [130].

### 3.4. The Interplay Between the Vaginal Microbiome and the Immune Microenvironment in HPV-Induced Lesions

As previously mentioned, vaginal dysbiosis can influence the host’s immune response and cause disruption of the defense barriers, thereby causing chronic infection and the release of pro-inflammatory cytokines which can ultimately lead to cervical (pre)malignant lesions [37].

In addition to *Lactobacillus*’ ability to maintain the acidic environment and low vaginal pH, it can produce metabolites and stimulate immune cells to release cytokines, thereby enhancing the host’s anti-infective response [131]. In VMB depleted of lactobacilli, the vaginal immune barrier is compromised, allowing HPV and other pathogenic microorganisms to adhere. This vaginal dysbiosis leads to a decrease in an effective cervical immune response while simultaneously promoting the colonization of abnormal vaginal flora [132]. VMB characterized by an abnormal pH, vaginal dysbiosis, and *Lactobacillus*-depletion showed elevated immune markers in vaginal lavages [41]. An increase in pro-inflammatory cytokines, IL-1β, IL-15, and TNF-α was observed. As well as an increase in regulatory cytokine IL-12, and growth factor FGF2 [41]. Transient inflammation is likely essential for clearance, but the presence of both pro-inflammatory and anti-inflammatory cytokines is indicative of chronic infection [47]. In another study, *Lactobacillus* spp. were negatively correlated with Th2 cytokine expression (IL-5, IL-13) and positively correlated with Th1 cytokine expression (IL-2 and IL-12) in cervicovaginal secretions [133]. IL-2 and IL-12 play a role in the survival and activation of T lymphocytes [134]. However, the various *Lactobacillus* spp. can differ in their immunoregulatory functions. For instance, *L. gasseri* (CST II) is associated with increased levels of IFN-γ and decreased levels of IL-17 in peripheral blood, whereas *L. iners*-dominance (CST III) is linked to optimal levels of both cytokines. IL-17 is produced by Th17 cells, which are a subtype of CD4+ T cells [128]. Remarkably, *G. vaginalis* (CST IV) is associated with elevated levels of IL-17 and reduced levels of IL-10 and IFN-γ in peripheral blood compared to *Lactobacillus*-dominated VMB. This negative correlation between IL-17 and IFN-γ suggests that bacteria associated with vaginal dysbiosis compromise the Th1 response by promoting the differentiation of Th17 cells and increasing IL-17 concentrations in the immune environment, thereby creating an ineffective antiviral immune response [128]. The presence of *Gardnerella* was positively correlated with Th2 cytokine expression and negatively correlated with Th1 cytokine expression in cervicovaginal secretions [133]. Additionally, enhanced levels of Toll like receptor (TLR) 7 and TLR 9 were found in HPV-infected cervical cells of BV-positive women [91]. Activation of TLRs leads to the production of IFNs and inflammatory cytokines. However, prolonged TLR activation might cause tissue damage [91]. Di Paola et al. associated *Prevotella* (CST IV) with HPV infection and persistence [94]. It can modulate the host’s mucosal immunity by increasing the number of cytokines, e.g., Th2-induced IL-5, in cervicovaginal samples [135] and by secreting proteases that degrade host antibodies and transfer ammonia to *Gardnerella* [136]. Additionally, *Prevotella* is positively correlated with IP-10 [137]. Chemokine IP-10 is an IFN-γ-induced protein involved in transporting immune cells to inflammatory sites [138]. *Lactobacillus*, on the other hand, inhibits IP-10 secretion and reduces the inflammatory response [139,140]. In VMB dominated by BV-associated bacteria, levels of IL-1β and TNF-α are increased in cervical secretions, which is associated with an increased risk of lesion progression [141]. *F. vaginae* (CST IV) is able to activate TLR 2 and NF-κβ in cervicovaginal epithelial cells, thereby triggering the production of inflammatory cytokines and Th1 IFNs, initiating an innate immune response [142,143]. Persistent activation of inflammatory factors can lead to chronic inflammation, which in turn facilitates the entry of HPV capsids and the development of cervical dysplasia [37].

Other than BV-associated bacteria, it is known that *C. trachomatis* (CST IV) can adhere to the genital mucosa and disrupt the epithelial barrier, thereby causing an inflammatory reaction and increasing the vaginal pH, which has a detrimental effect on cervicovaginal immunity and facilitates the invasion of other pathogenic microorganisms. This increases the risk of a persistent HPV infection [144].

*Atopobiaceae* (CST IV) were enriched in cLSIL, cHSIL, and cervical carcinoma and positively correlated with IL-1α, IL-4, IL-10, IL-12, IL-36γ, IFN-α2, IFN-γ, and TNF-α in cervicovaginal lavages. *Atopobiaceae* were negatively correlated with IP-10 and CCL20 [89]. CCL20, also known as MIP-3α, is a chemokine involved in attracting immune cells to the inflammation site [145].

*Fusobacterium* spp. (CST IV) were associated with higher levels of cervical IL-4 and TGF-β1 [40].

In conclusion, lactobacilli enhance pro-inflammatory immunity by promoting Th1 cytokine expression and decreasing Th2 cytokine expression [133]. These characteristics are typical of transient infections, while a higher alpha diversity and abundance of BV- and CST IV-associated bacteria contribute to chronic infection by increasing pro-inflammatory and anti-inflammatory cytokines in the cervicovaginal environment. As reviewed by Muntinga et al., cervical intralesional immunity is believed to play a role in protecting against HPV infections as well [15]. However, current literature mostly describes changes in immune factors within the cervicovaginal space, and the precise interplay between the VMB, vaginal immunity, and cervical immunity remains unclear. It is plausible that altering the VMB to a more optimal, *Lactobacillus*-dominant environment could help in creating an effective immune response to clear HPV infections and promote cervical lesion regression.

### 3.5. Influence of Treatments for cHSIL on the Vaginal Microbiome and Immunological Response

The treatment of cervical intraepithelial neoplasia (CIN) depends on the severity of the lesion. cLSILs spontaneously regress in 60% of patients [146] and expectant management is preferred. In contrast, cHSILs have lower regression rates and more often necessitate treatment. Expectant management may be considered for women under 30 years with CIN 2, as 55% of cases regress spontaneously [146]. Patients with CIN 3 generally need treatment as only 28% spontaneously regress [146]. The most common treatment is a large loop excision of the transformation zone (LLETZ) [147]. While it is effective in up to 90% of patients, complications such as hemorrhage and an almost doubled risk of premature birth occur [147,148,149]. Therefore, alternative treatment strategies are warranted. Imiquimod, a topical immunomodulatory cream that stimulates the immune response by binding to Toll-like receptors (TLRs) 7 and 8 on immune cells, has been studied as an alternative treatment option. However, response rates vary between 52% and 73% [150,151,152]. In this section, we summarize current knowledge on the effects of different treatments on VMB composition and host immunity in HPV infection and outline future directions for alternative therapies in cSILs.

#### 3.5.1. Effects of LLETZ on VMB and Immunity

Across different populations, LLETZ resulted in shifts towards a *Lactobacillus*-dominant VMB. Zhang, et al. observed a transition from CST IV (*Prevotella* spp. and *Sneathia* spp.) to CST III (*L. iners*) 3 months post-LLETZ (*n* = 26) [153]. This transition occurred simultaneously with hrHPV clearance. Similarly, Giovannetti et al. found a significant decrease in CST IV-related *Streptococcus* and *Prevotella* 3 months post-LLETZ, with *U. parvum* and *Streptococcus* spp. remaining significantly reduced after 6 months (*n* = 23) [154]. While Wiik et al. and Zhang et al. reported increased *Lactobacillus* spp. abundance at 3 and 6 months [153,155], no significant changes were found at 12 months [155]. Caselli et al. (*n* = 85) found a significant increase in *L. crispatus* (CST I) and a decrease in CST IV bacteria at least 6 months post-LLETZ in patients who cleared hrHPV, whereas those with persistent hrHPV infection showed no significant changes in VMB composition [156]. Similarly, Kawahara et al. (*n* = 41) found a decrease in *Atopobium* spp. (CST IV) in patients who cleared HPV post-LLETZ [157].

To our knowledge, there are few studies investigating the interplay between the VMB and the immune microenvironment in HPV-induced lesions pre- and post-treatment. Pro-inflammatory cytokines (e.g., IL-1α, IL-1β, IL-6, IL-8, TNF-α) in the vaginal environment were significantly reduced post-LLETZ in HPV-cleared patients. These levels remained higher in patients with HPV persistence [156,157].

#### 3.5.2. *Lactobacillus* as Probiotics

Many strategies have been suggested to improve the VMB, the most well investigated being probiotics. Given its role in promoting a healthy VMB, *L. crispatus* may have a potential in cSIL treatment. Oral administration of *L. crispatus* M247 in 24 CST IV patients with HPV infection resulted in *L. crispatus* dominance in 23 patients post-treatment with only 20% of these patients being HPV-positive [158]. Dellino et al. (*n* = 160) showed that HPV-positive patients with cLSIL treated with oral *L. crispatus* M247 during 12 months, showed higher lesion regression rates and HPV clearance compared to the HPV-positive control group [159]. In 50 hrHPV-positive patients, intravaginal transplantation of *L. crispatus* led to a significant increase in HPV clearance and restoration of *Lactobacillus* abundance after 6 months compared to the control group [160].

#### 3.5.3. Immunomodulatory Treatment

While the use of probiotics is an emerging field, no studies have investigated the effects of immunomodulatory treatment, including imiquimod, on the VMB. Previous research has shown that imiquimod induces a strong influx of intraepithelial and stromal CD4+ T cells in patients who responded to imiquimod treatment for cHSIL, but not in non-responders. It is believed that a ‘hot’, pro-inflammatory, cervical immune microenvironment is necessary for a complete response to imiquimod therapy [15,161]. However, its relationship with the VMB remains unknown.

In conclusion, previous research indicates that LLETZ generally promotes a shift towards a healthier, *Lactobacillus*-dominant VMB, and a reduction in vaginal inflammation, coinciding with HPV clearance. In patients with HPV persistence, however, the reverse is seen with the persistence of dysbiosis, chronic vaginal inflammation, and a dysfunctional systemic immunity against HPV. Probiotics aid in restoring a healthy VMB, resulting in improved HPV clearance in cLSIL, however little is known of its effect in the treatment of cHSIL. Imiquimod can be used as a noninvasive treatment of cHSIL, however, a pre-existing functional cervical microenvironment seems necessary for lesions to regress. It could be hypothesized that treatment of vaginal dysbiosis could result in restoration of a more functional cervical immunity, facilitating response to imiquimod.

## 4. Discussion

In this narrative review, we summarize current literature on the interplay between the vaginal microbiome and the immune microenvironment in cervical carcinogenesis. Research has shown that a *Lactobacillus*-dominated VMB is beneficial, with *L. crispatus* (CST I) being the optimal community, as it is most commonly associated with HPV-negative outcomes [43,51,52], HPV clearance [53], and cervical lesion regression [55]. This protective state is likely related to *Lactobacillus’* ability to maintain an acidic environment and preserve the mucosal barrier, preventing pathogens from invading epithelial cells [131]. Additionally, lactobacilli can produce metabolites and stimulate immune cells to release pro-inflammatory cytokines such as IL-1β and TNF-α [41]. Vaginal dysbiosis, characterized by greater diversity, is associated with higher susceptibility to HPV infection and persistence [51], as well as an increased risk of cervical dysplasia [103], with diversity rising as lesions progress [50,162]. Increasing diversity is associated with higher levels of inflammatory cytokines [78], indicative of a chronic inflammatory status impairing host immunity [109].

HPV capsids are able to modulate the host’s immune response by upregulating TLR activation and cytokine production. However, additional factors are required to drive cervical dysplasia progression. Next to HPV’s immune-evading strategies, vaginal dysbiosis can lead to changes in the host’s immune response, further altering the cervicovaginal environment and contributing to cervical carcinogenesis [78].

LLETZ has been associated with a shift towards a *Lactobacillus*-dominant VMB in HPV-cleared patients. In contrast, HPV persistence post-LLETZ is related to vaginal dysbiosis and chronic inflammation [153,154,155,156,157]. While probiotics have been shown to alter the VMB to a more optimal state in cLSIL [158,159,160], its effects in cHSIL remain unexplored. Imiquimod is a noninvasive, alternative treatment strategy for cHSIL. However, a pre-existing functional cervical immune microenvironment appears to be crucial for a complete response [161]. Restoring vaginal dysbiosis, and consequently promoting a more balanced cervical immune state, could enhance the efficacy of imiquimod therapy in cHSIL patients.

As study selection and screening were performed by a single reviewer, this may have introduced a degree of selection bias, which should be considered when interpreting the findings.

## 5. Conclusions

To conclude, the VMB plays a pivotal role in the acquisition and persistence of hrHPV infection and cervical carcinogenesis. An optimal VMB is characterized by a high abundance of *Lactobacillus* spp., particularly *L. crispatus*, and low microbial diversity. This is associated with HPV-negative outcomes and a healthy cervix. In contrast, communities dominated by anaerobic bacteria, with high diversity, are detrimental to cervical health. Especially BV-associated bacteria are linked to a higher risk of HPV acquisition and progression of cervical lesions. While *Lactobacillus* spp. promote the production of cytokines associated with transient infections, bacteria linked to vaginal dysbiosis produce high levels of cytokines associated with chronic inflammation, increasing the risk of progression to cervical carcinoma. Understanding the interplay between the VMB and immune responses may be key to developing signatures that can aid in predicting treatment response and developing new treatment strategies.

## 6. Future Directions

To reduce the need for invasive diagnostics and treatments in HPV-positive patients, identifying biomarkers, microbial and/or immunological, is essential to determine which women are likely to experience spontaneous regression and which will not. Moreover, the identification of biomarkers will aid in identifying new therapeutic strategies targeting the VMB, the immunity, or both, to ensure that women receive the most appropriate therapy for their individual status and to avoid unnecessary LLETZ procedures, associated with risks of preterm birth.

## Figures and Tables

**Table 1:** Overview of vaginal community state types.

Community State Type (CST)	Dominant Species
CST I	*Lactobacillus crispatus*
CST II	*Lactobacillus gasseri*
CST III	*Lactobacillus iners*
CST IV	Diverse bacteria, not dominated by *Lactobacillus*
CST V	*Lactobacillus jensenii*

## Supplementary Materials

The following supporting information can be downloaded at: https://www.mdpi.com/article/10.3390/ijms26093954/s1.

## Abbreviations

The following abbreviations are used in this manuscript:

BV	Bacterial vaginosis
CIN	Cervical intraepithelial neoplasia
cHSIL	Cervical high-grade squamous intraepithelial lesion
cLSIL	Cervical low-grade squamous intraepithelial lesion
cSIL	Cervical squamous intraepithelial lesion
CST	Community state type
HPV	Human Papillomavirus
hrHPV	High-risk Human Papillomavirus
LLETZ	Large loop excision of the transformation zone
VMB	Vaginal microbiome
