# Supplementary material for: Vaginal Microbiota and Local Immunity in HPV-Induced High-Grade Cervical Dysplasia: A Narrative Review
Source: Int J Mol Sci. 2025 Apr 22;26(9):3954. doi: 10.3390/ijms26093954 (PMC12071600; doi:10.3390/ijms26093954)
Supplement: Supplementary file 1 [file ijms-26-03954-s001.zip › ijms-3567097-supplementary.pdf]

Supplementary Materials

Table S1. Search strategy

**Database Searched**	**Date**	**Search Terms**	**Search Results**
**PubMed**	January 29, 2025	((("cervi*"[Title/Abstract] OR "vagina*"[Title/Abstract] OR "cervicovagina*"[Title/Abstract]) AND ("microbio*"[Title/Abstract] OR "dysbiosis"[Title/Abstract] OR "dysbacteriosis"[Title/Abstract] OR "flora"[Title/Abstract] OR "dysbiosis"[MeSH Terms] OR "microbiota"[MeSH Terms:noexp])) OR "Lactobacillus"[MeSH Terms] OR "Lactobacillus"[Title/Abstract] OR "vagina/microbiology"[MeSH Terms:noexp]) AND ("uterine cervical dysplasia"[MeSH Terms] OR "uterine cervical neoplasms"[MeSH Terms] OR "CIN"[Title/Abstract] OR "cervical intraepithelial neoplasia"[Title/Abstract] OR "HSIL"[Title/Abstract] OR "high grade squamous intraepithelial lesion"[Title/Abstract] OR "hpv"[Title/Abstract] OR "human papillomavirus"[Title/Abstract] OR "squamous intraepithelial lesions"[Title/Abstract] OR "SIL"[Title/Abstract] OR ("Atypical Squamous Cells"[Title/Abstract] AND "cervix"[Title/Abstract]))	883
**Embase (OVID)**	January 29, 2025	((("cervi*" or "vagina*" or "cervicovagina*").ti,ab,kf. and (("microbio*" or "dysbiosis" or "dysbacteriosis" or "flora").ti,ab,kf. or "dysbiosis"/ or "bacterial flora"/ or "bacterial microbiome"/ or "microbiome"/)) or "Lactobacillus"/ or "vagina flora"/ or "Lactobacillus".ti,ab,kf.) and (exp uterine cervix dysplasia/ or exp cervical intraepithelial neoplasia/ or "squamous intraepithelial lesion of the cervix"/ or ("CIN" or "cervical intraepithelial neoplasia" or "HSIL" or "high grade squamous intraepithelial lesion" or "hpv" or "human papillomavirus" or "squamous intraepithelial lesions" or "SIL" or ("Atypical Squamous Cells" and "cervix")).ti,ab,kf.)	1327
